# Development and validation of a distributed representation model of Japanese high-dimensional administrative claims data for clinical epidemiology studies

**DOI:** 10.1186/s12874-025-02549-7

**Published:** 2025-04-11

**Authors:** Hiroki Matsui, Kiyohide Fushimi, Hideo Yasunaga

**Affiliations:** 1 Department of Clinical Epidemiology and Health Economics, School of Public Health, The University of Tokyo, 7-3-1 Hongo, Bunkyo-Ku, Tokyo, 1130033 Japan; 2 Department of Health Policy and Informatics, Institute of Science Tokyo Graduate School of Medical and Dental Sciences, 1-5-45 Yushima, Bunkyo-Ku, Tokyo, 1138519 Japan

**Keywords:** Distributed representation, Word2vec, Administrative claims data, High-dimensional propensity score, Unmeasured confounder

## Abstract

**Background:**

Unmeasured confounders pose challenges when observational data are analysed in comparative effectiveness studies. Integrating high-dimensional administrative claims data may help adjust for unmeasured confounders. We determined whether distributed representations can compress high-dimensional administrative claims data to adjust for unmeasured confounders.

**Method:**

Using the Japanese Diagnosis Procedure Combination (DPC) database from 1291 hospitals (between April 2018 and March 2020), we applied the word2vec algorithm to create distributed representations for all medical codes. We focused on patients with heart failure (HF) and simulated four risk-adjustment models: 1, no adjustment; 2, adjusting for previously reported confounders; 3, adjusting for the sum of distributed representation weights of administrative claims data on the day of hospitalisation (novel method); and 4, a combination of models 2 and 3. We re-evaluated a previous study on the effect of early rehabilitation in patients with HF and compared these risk-adjustment methods (models 1–4).

**Results:**

Distributed representations were generated from the data of 15 998 963 in-patients, and 319 581 HF patients were identified. In the simulation study, Model 3 reduced the impact of unmeasured confounders and achieved better covariate balances than Model 1. Model 4 showed no increase in bias compared with the true model (Model 2) and was used as a reference model in the real-world application. When applied to a previous study, models 3 and 4 showed similar results.

**Conclusion:**

Distributed representation can compress detailed administrative claims data and adjust for unmeasured confounders in comparative effectiveness studies.

**Supplementary Information:**

The online version contains supplementary material available at 10.1186/s12874-025-02549-7.

## Background

The increasing accessibility of large-scale medical observational data have enabled comparative effectiveness studies. As administrative claims data comprise large-scale medical observational data, mostly in a standard format, their value in comparative effectiveness studies has increased. However, the lack of patient severity information and the resultant unmeasured confounders are a major drawback of administrative data [1] that constitute a limitation in many epidemiological studies [2, 3]. Thus, considerable effort has been expended to collect detailed information on confounders from non-administrative data sources, such as electronic medical records [4, 5].

Unmeasured confounders have been addressed previously through various approaches. If appropriate variables are present, the instrumental variables (IV) method can adjust for unmeasured confounders [6–9]. However, optimal IV are not always available; in fact, situations where appropriate IVs exist are rather rare. Furthermore, the estimated effect represents the Local Average Treatment Effect (LATE), which is more challenging to interpret compared to measures such as the Average Treatment Effect (ATE) that is calculated using propensity scores. In contrast, Schneeweiss et al. proposed the high-dimensional propensity score (HDPS) to calculate propensity scores from a large number of variables and adjust for unmeasured confounders by using them as proxy variables [10, 11].

When we focus on a different domain, matching similar documents with a huge vocabulary is being challenged in the field of natural language processing [12]. For example, Mikolov developed the word2vec method, which embeds a high-dimensional vocabulary within a fixed k-dimensional matrix using a distributed representation of words [13]. This method enables the calculation of the meaning of inter-word distances and has been used to match document tasks in the social sciences [14] and medical research [15–18]. Though Weberpals et al. proposed the application of a deep-learning-based autoencoder to embed high-dimensional information and risk adjustment [19], there exists a knowledge gap whether embedding high-dimensional information can contribute to the adjustment for unmeasured confounders by using this embedded high-dimensional information as proxy variables of unmeasured confounders. We proposed a novel method to adapt distributed representations of medical records to large-scale, multidimensional administrative data to achieve propensity balancing and conduct comparative effectiveness studies.

This study aimed to investigate whether embedded medical information could be used to perform risk adjustments for administrative claims data. Specifically, we developed distributed representations of administrative claims data and evaluated whether the novel method could reduce bias due to unmeasured confounders through a simulation study using real-world data. Finally, we adapted our new method to a previous clinical epidemiology study that examined whether early initiation of rehabilitation in patients with heart failure (HF) could improve outcomes [20].

## Methods

### Development of distributed representation

The first step in our research was to develop a distributed representation of all codes within the Japanese Diagnosis Procedure Combination (DPC) database, the largest Japanese nationwide inpatient database the comprises data from 1291 hospitals. For a distributed representation, we applied the word2vec algorithm of Mikolov et al. (2013) [13] to all inpatient records in DPC data between 1 April 2018 and 31 March 2020 (details in Supplement 1) that contained administrative data and disease-specific severity information (Supplement 1). To apply the word2vec algorithm to non-natural language data, we referred to the Medical Concept Embeddings from Medical Claims (MCEMC) proposed by Choi et al. [16]. The details of the distributed representation learning process are described in Supplement 2.

### Data source for a simulation study and application to real-world data

With reference to a previous study [20], we extracted the records of adult in-patients with HF. The inclusion criteria were patients: 1) with an HF diagnosis (ICD10: I50$), 2) aged > 20 years, and 3) discharged during the study period. The exclusion criteria were patients: 1) discharged on the first day of hospitalisation, 2) undergoing major surgery under general anaesthesia, 3) receiving mechanical ventilation, intra-aortic balloon pumping, or extracorporeal membrane oxygenation on the first day of admission, and 4) who started rehabilitation on the first day of admission.

#### Study variables

Referring to a previous study [20], we collected the following patient information from the DPC database: background information, diagnoses at admission, medications prescribed, and procedures performed on the first day of admission. We obtained detailed severity information for HF patients from discharge summary records and used the diagnosis and all medical practice information from the first day of admission (study variables are described in Supplement 3).

#### Using distributed representation for risk adjustment

We used a distributed representation for risk adjustment as follows: We obtained a code list (keys) for each patient’s diagnostic information (diagnosis at admission and comorbidity diagnosis) and medical treatment (medications, procedures, and medical supplies) on the first day of hospitalisation. Second, fixed length numeric value vectors (weights of diagnosis or medical treatment administrative codes) were assigned to these codes. Third, the sum of the weights for each patient was computed. Finally, we used the sum as a covariate to determine the propensity score. We assigned weights to the diagnosis and medical treatment administrative codes such that codes with stronger relationships would be positioned closer together in a 200-dimensional space. Consequently, by summing these weights (i.e., taking the vector sum), we could assign 200-dimensional vectors to patients whose initial diagnoses and treatments were similar, ensuring that vectors for comparable patients lay near one another. Thereafter, we treated each patient’s resulting 200-dimensional vector as a variable representing their overall initial diagnosis and medical treatment status.

We compared the following four risk-adjusted models: 1 (no risk adjustment and risk differences were obtained), 2 (propensity score matching based on the variables in a previous study, including detailed severity information of HF patients), 3 (propensity score matching using the sum of embedding weights, with age and sex), and 4 (merging variables from models 2 and 3). We included a 200-dimensional vector, representing each patient’s initial diagnosis and medical treatment status on the day of admission, as a covariate in Models 3 and 4.

We performed 1:1 propensity score matching of treatment and control group, using the nearest-neighbour method with a calliper of 1% standardised deviation.

### Simulation study

We conducted a simulation study to validate the efficacy of our method for minimising the unmeasured confounding biases. Using random treatment indicators and weighted sampling, we created a dataset that mimicked the confounding effects and tested each risk-adjustment model to determine whether it could correct for confounding effects.

The simulation process is as follows: First, we generated a random treatment-assignment indicator to ensure that there was no association between the prognosis and treatment assignment (risk difference = 0).

Second, we constructed a prognostic model using variables, including disease severity. We set the composite outcome of in-hospital death and dependency on activities of daily living (ADLs) at discharge as the prediction target outcome. The risk-adjustment method in Model 2 is the true risk-adjustment method used in this simulation study (the prognostic model is described in Supplement 4). The cohort was divided into quartiles of the predicted values (Q1-Q4). Third, equal sampling from groups with a 0 treatment indicator (from Step 1) was performed until 10,000 cases formed the control cohort. Fourth, for the treatment cohort, we used weighted sampling from the groups of Q1-Q4. For the cohort that was least likely to have an outcome, we sampled 40% of Q1, 30% of Q2, 20% of Q3, and 10% of Q4 (0.4, 0.3, 0.2, and 0.1, respectively). For the cohort most likely to have the outcome, we sampled 10% from Q1, 20% from Q2, 30% from Q3, and 40% from Q4 ( 0.1, 0.2, 0.3, and 0.4, respectively). We developed ten sampling scenarios between these extremes to create varying bias intensities.

Finally, the control and treatment cohorts were combined to form the simulation cohort, which was then risk-adjusted using models 1–4 to compare the intergroup differences in prognoses by treatment.

In this simulation, each model was defined as follows: Model 1 omitted all confounding variables from the true model (incomplete model); Model 2 was the true model (complete model); Model 3 omitted all confounding variables from the true model but applies a new adjustment method (proposed model); and Model 4 was the true model with the new adjustment method additionally applied. We aimed to confirm that Model 4 did not exhibit bias amplification when the variables used in Model 3 were added to Model 2 (true model).

This process was repeated 100 times for each scenario. For each variable, we calculated the intergroup risk differences with 95% confidence intervals (CI). As indicators of group balancing, we calculated standardised mean differences (SMD) for all variables used in the prognostic model in the second step and assessed the c-statistics for the treatments in Model 4 in the propensity-matched cohort. On achieving the covariate balance with matching, the c-statistic of the matched cohort was 0.5. We summarised the medians and CI of the risk differences and probability of coverage with 95% CI, SMD, and c-statistics.

### Real-world data application

We evaluated early initiation of mobilisation in patients hospitalised with heart failure as described previously [20] and early initiation of rehabilitation in in-patients with HF after adjusting for disease severity using both administrative and detailed patient-severity information included in the DPC database.

#### Exposure definition

Patients who began rehabilitation within 2 days of hospitalisation were categorised into the exposure group, and those starting after the third day were categorised as the control group.

#### Outcome variables

The primary outcome was a combination of in-hospital death and ADLs dependency at discharge, measured using a Barthel Index ≤ 60. The secondary outcomes included in-hospital mortality, combined in-hospital death, 90-day post-discharge readmission, and length of hospital stay.

### Statistical analysis

Descriptive statistics of patient information for the early and delayed rehabilitation cases were obtained for the pre-matching cohort. The intergroup balance was evaluated using SMD [21]. We calculated the differences in outcomes between the control and exposure groups. CI were obtained using the bootstrap method.

### Bootstrap simulation

The estimated risk differences and balance measures for each adjustment method were compared with those of real-world data bootstrap simulations. We performed 1000 bootstrap simulations. Owing to computational constraints, each bootstrap cohort was drawn from a randomly chosen 20% of the study cohort.

To evaluate covariate balancing in each model, we calculated the SMD of each variable and the c-statistics of Model 4 in the propensity score-matched cohorts. To evaluate the validity of these risk-adjustment methods, we obtained the intergroup difference in outcome for each risk-adjustment method. We calculated the ‘CI width of the results’ as a measure of efficiency and compared these results with those obtained using Model 4. We calculated 95% bootstrap CI for these measures.

## Results

### Embedding with distribution representation

We obtained records of 15 998 963 patients discharged between April 2018 and March 2020 and 77 364 dimension tokens from the DPC data and used word2vec to obtain distribution representation weights with 200 dimensions. All weights are publicly available on the authors’ website [22].

### Data extraction the simulation study and real-world data application

**Fig. 1:**
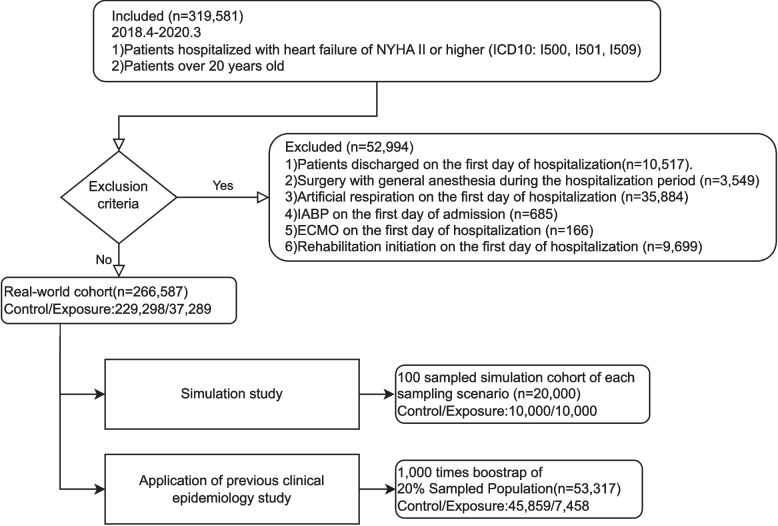
Patient selection flowchart

We extracted DPC data for HF cases (319 581 cases) that met the inclusion criteria and were discharged between April 2018 and March 2020 (Fig. 1). We excluded 52,994 patients that met the exclusion criteria, leaving 266 587 patients in the real-world cohort (229 298 and 37 289 patients with delayed and early rehabilitation, respectively). The administrative information for the real-world cohort contained 8408-dimensional tokens and patient demographics are shown in Supplementary Table S1. Systolic blood pressure, hypertension diagnosis, and intravenous furosemide, carperitide, and tolvaptan use were imbalanced between groups.

### Simulation study

A prognostic model was constructed prior to the simulation. The constructed prognostic model had a high discriminative power (area under the curve: 0.868) and good calibration (Supplement 4). Simulation was performed using the constructed prognostic model.

**Fig. 2:**
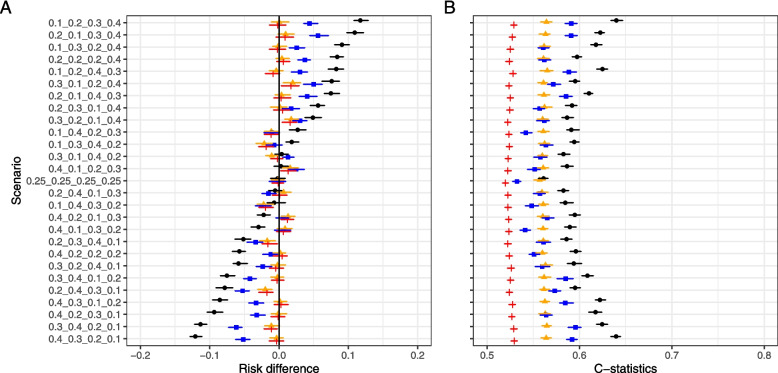
The results of the simulation in each scenario in the four risk-adjustment models. **A,** distribution of the estimated risk differences in each simulation scenario; **B**, the covariate balances between the exposure and control groups evaluated using c-statistics of the matched cohort in each scenario. Dots indicate point estimates and bars indicate 95% confidence intervals. The dots and bars are black, orange, blue, and red for models 1, 2, 3, and 4, respectively

**Fig. 3:**
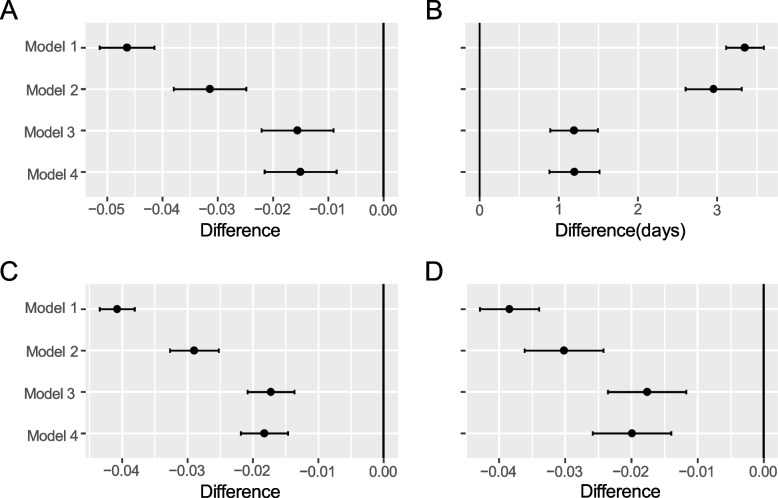
Estimated risk differences in early mobilization for primary and secondary outcomes in each risk-adjustment model. Panel **A**: In-hospital death and dependency (Barthel Index < 60) on activities of daily living. Panel **B**: In-hospital mortality. Panel **C**: Composite outcomes of in-hospital death and readmission within 90 days. Panel **D**: Length of hospital stay

Figures 2A and 3 shows the distribution of the estimated risk differences in each simulation scenario for the four risk-adjustment methods. Though assumed to be 0, the unadjusted risk difference in Model 1 (depicted with black dots and bars) showed wide variation among the different sampling scenarios; Models 2–4 showed a relatively unbiased risk difference compared with Model 1 in all scenarios; Model 3 showed a smaller risk reduction for the true risk difference (risk difference = 0) than Models 2 and 4 and showed similar risk differences as Models 2 and 4 in small-bias (within about 5% biased risk-difference) scenarios; and Model 4 demonstrated a risk difference equivalent to that of the true model (Model 2), with no observed increase in bias. The probabilities of covering the true values of the CI for models 1–4 were 17.8%, 72.3%, 24.0%, and 75.5%.

Figure 2B shows the covariate balances between the exposure and control groups evaluated using the c-statistics of the matched cohort in each scenario in the four risk-adjustment models. Models 2 and 3 showed improvements in balance compared to Model 1. Each covariate balance assessed with SMD improved even when the unadjusted imbalance was greater than the other covariates, such as age, Japan Coma Scale (JCS) score, and ADLs on admission (Supplement 5).

### Real-world data application

#### Covariate balance in each risk-adjustment model

Supplementary Figure S1 shows the covariate balance for each risk-adjustment model measured using SMD. Models 2 and 3 showed improvements in balance compared with Model 1, without covariate imbalances. Supplementary Figure S3 shows the estimated risk differences in early mobilization for primary and secondary outcomes in each risk-adjustment model. Compared to Model 1, early rehabilitation had a smaller estimated risk difference in models 2–4. Models 3 and 4 exhibited similar risk differences.

#### Bootstrap simulation

We obtained data for 13 676 patients (control/exposure: 11 857/1 819), representing 5% of the sampled population from a real-world cohort.

#### Balance of each bootstrap cohort

The SMD for each variable is shown in Supplementary Figure S2. Models 2 and 3 showed improvements in balance compared with Model 1, without covariate imbalances.C-statistics of propensity score-matched cohorts.

We evaluated the covariate balances between the exposure and control groups using the c-statistics of the matched cohort in each model. Model 4 (0.555 [95% CI, 0.549–0.561]) was the most balanced, followed by models 3 (0.579 [0.571–0.587]), 2 (0.690 [0.681–0.698]), and 1 (0.710 [0.704–0.716]).

#### Bias of estimated outcome measures

**Fig. 4:**
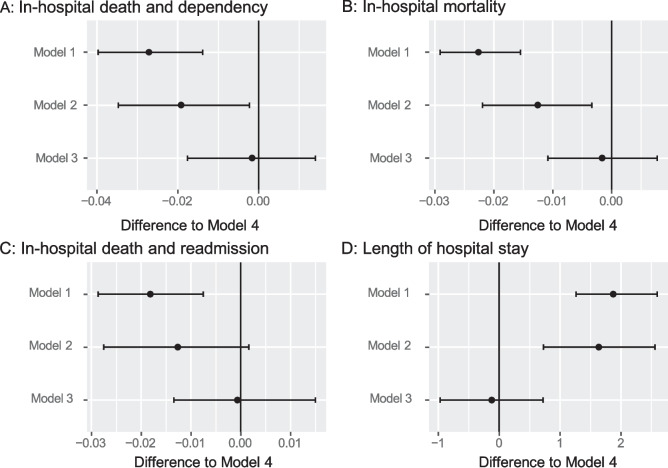
Differences of estimated outcome measures in models 1–3 compared with Model 4 (propensity score matching using variables included in models 2 and 3).** A**, In-hospital death and dependency (Barthel Index < 60) on activities of daily living; **B,** In-hospital mortality; **C**, Composite outcomes of in-hospital death and readmission within 90 days; and **D**, Length of hospital stay

Using bootstrap samples, we compared the estimated outcome measures obtained from models 1–3 with those from Model 4. No difference was observed between models 3 and 4 (Fig. 4).

#### Width of obtained confidence intervals

**Fig. 5:**
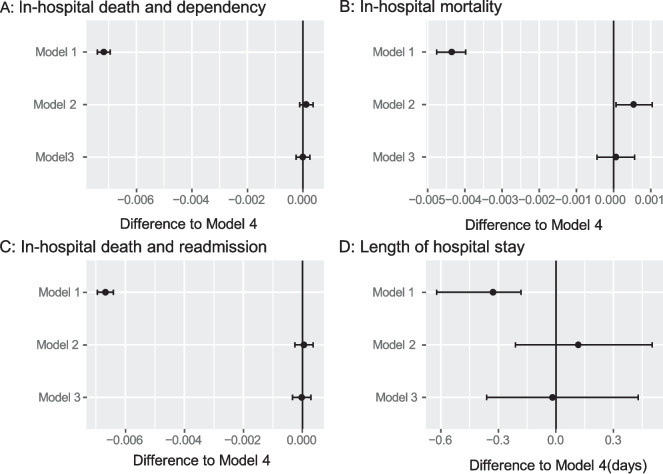
Confidential interval width for models 1–3 compared to Model 4 based on bootstrap simulation results. **A**, In-hospital death and dependency (Barthel Index < 60) on activities of daily living; **B**, In-hospital mortality; **C**, Composite outcomes of in-hospital death and readmission within 90 days; **D**, Length of hospital stay

Using bootstrap samples, we compare the widths of the CI obtained from models 1–3 with those from Model 4. No significant difference was observed, except in-hospital mortality, between models 2 and 4 (Fig. 5).

## Discussion

In this study, we developed and utilised distributed representations of medical records from the DPC database to develop a novel risk-adjustment model that compressed the 77 364-dimensional data into 200 dimensions. Various methods have been proposed for embedding non-linguistic information using vector representations. Choi et al. constructed an MCEMC based on the medical claims information of approximately 4 million people [16]. Nelson et al. used the PageRank algorithm to embed patient information from unstructured electronic health records into a biomedical knowledge graph that was subsequently used to predict multiple sclerosis [23, 24]. We used the method proposed by Choi et al. [16] to embed Japanese medical claims data. The distributed representation weights obtained in this study represent all codes in the Japanese medical claims data as a vector: HF (I50), the target disease of this study, is related to various drugs and medical procedures (Supplement 6).

We tested the new risk-adjustment model using real-world data to investigate whether it could reduce the bias from unmeasured confounders in a simulation study and showed that the new model (Model 3) effectively achieved this and improved covariate balances. Model 4, combining variables from Model 3 and the true risk-adjustment model (Model 2), showed no increase in bias compared with the true model (Model 2). That is, we confirmed that Model 4 did not exhibit a bias amplification when the variables used in Model 3 were added to Model 2 (true model). Thus, Model 4 was used as the reference risk-adjustment model for real-world applications.

We compared the results of our new risk-adjustment model with those of a previous study [20]. Model 3 obtained a better balance than models 1 and 2 whereas models 3 and 4 yielded similar results.

In clinical epidemiological studies, addressing unmeasured confounders is crucial for the accurate estimation of effectiveness. The IV method was used to mitigate unmeasured confounders [6–9], wherein hospital rehabilitation preference was used as an IV and confirmed the robustness of the results for the primary outcome [20]. Questions regarding the validity of these variables persist because of instrumental outcome confounders. Furthermore, optimal IV are not always available.

The HDPS proposed by Schneeweiss et al. uses numerous variables, including proxy variables, to adjust for unmeasured confounders [10, 11]. Recent advancements have incorporated deep learning and LASSO regression into HDPS computation [1, 25]. Weberpals et al. conducted risk adjustment using deep-learning-based embedding [19].

We reduced bias from unmeasured confounders by incorporating comprehensive medical data into our model and using them as proxy variables. Using the derived distributed representation weights, we depicted treatments and the diagnosis on the first day as a 200-dimensional numeric vector. Because treatments are decided based on patient severity, the initial treatment may have acted as a proxy for unrecorded severity, such as left ventricular ejection fraction.

Though the JCS and ADLs at admission were not treatments, the simulations indicated an improved covariate balance for these variables. The enhanced balance suggests that the proposed methodology may feasibly adjust for several crucial covariates besides the JCS and ADLs at admission. The simulation results indicated that adjusting for all confounders and medical practice information on the first day of admission did not bias the estimated treatment effect. Thus, Model 4’s estimation results minimally influenced unmeasured or neglected confounders. In real-world data applications, models 3 and 4 had similar risk differences. Therefore, Model 3 was considered to estimate the treatment effects closely and accurately, even in real-world data with unmeasured or neglected confounding.

Model 2 showed more biased results compared to Model 3 and Model 4 in real-world applications. This suggests that the risk adjustment in Model 2 might be insufficient owing to the influence of unmeasured confounders or the omission of detailed treatment information from the model, as commonly observed in traditional studies. The proposed Model 3 demonstrated the potential to perform risk adjustment beyond capabilities of expert knowledge, indicating its contribution to the standardization and simplification of epidemiological research using large-scale data. The findings of this study offer advantages over previous methods. Weberpals et al. utilised a deep-learning-based autoencoder for embedding [19] and HDPS [1]. This study used a novel method for simplifying high-dimensional covariates using a database. All the codes were assigned a specific value for the representation weight to enable simple calculation of the sum of representation weights.

HDPS, compared to the proposed method, identifies factors that may influence bias by analyzing their relationships with outcomes and exposures. In contrast, the proposed method calculates weights using an unsupervised learning approach, which does not require the explicit definition of outcomes or exposures. This flexibility allows precomputed weights to be applied to various epidemiological challenges, highlighting high versatility in the proposed method. In future research, this approach could be expanded by embedding the quantized data method used in this study for confounding adjustment. Such advancements could enable the incorporation and analysis of a broader range of data types, including natural language and image information to the model, further enhancing its applicability.

The methods used in this study can be readily applied in clinical epidemiological research. The distributed representation weight table, which allows researchers to convert medical records into 200-dimensional features, is accessible from the author’s website. This approach is especially beneficial for large databases, such as Japan's National Database of Health Insurance Claims, which lacks detailed patient data but offers comprehensive coverage of the population [26], whereby researchers can bolster comparative effectiveness research using these databases.

This study had some limitations. We ignored the amount of medicine in each prescription or combinations of diagnoses and treatments. Implementing more precise patient matching may be possible by applying network structures such as transformer [27], which have been used in natural language processing in recent years. In this study, the number of embedding dimensions is fixed at 200. Therefore, further studies are required to determine whether the same results can be obtained by varying the number of dimensions. Second, it is difficult to guarantee that the constructed distributed representation is the best representation model. Third, the method used in this study, unlike HDPS, cannot explicitly identify the factors that directly contribute to confounding adjustment. Fourth, while simulation experiments confirmed that there was no increase in bias in Model 4, which was subsequently used as the reference model in real-world data applications, there is no guarantee that Model 4 represents the true model.

## Conclusions

We demonstrated that embedded medical information can be used to perform risk adjustments for administrative medical data.

## Supplementary Information

Supplementary Material 1.

## Data Availability

The raw data supporting this study's findings are not publicly available at the time of submission because of restrictions imposed by the ethics committee with regard to privacy concerns. These data are available upon reasonable request from the corresponding author.

## Abbreviations

IV: Instrumental variables
LATE: Local Average Treatment Effect
ATE: Average Treatment Effect
HDPS: High-dimensional propensity score
HF: Heart failure
DPC: Diagnosis Procedure Combination
MCEMC: Medical Concept Embeddings from Medical Claims
ADLs: Activities of daily living
CI: Confidence intervals
SMD: Standardised mean differences
JCS: Japan Coma Scale
