# Supplementary material for: Development and validation of a distributed representation model of Japanese high-dimensional administrative claims data for clinical epidemiology studies
Source: BMC Med Res Methodol. 2025 Apr 11;25:95. doi: 10.1186/s12874-025-02549-7 (PMC11987422; doi:10.1186/s12874-025-02549-7)
Supplement: Supplementary file 1 — Supplementary Material 1. [file 12874_2025_2549_MOESM1_ESM.docx]

## Supplementary Materials

#### Supplement 1: Description of the DPC database

We used the Japanese Diagnosis Procedure Combination (DPC) database, the largest nationwide Japanese inpatient database comprising data collected from 1,291 hospitals. The DPC data contain both administrative and patient severity information [1]; the administrative data include patient demographics (age and sex), patient diagnosis information recorded according to the International Disease Classification Code 10th Revision (ICD10), diagnosis at admission, primary diagnosis, comorbidities, complications, and daily medical treatment process information (YJ code: original Japanese code of medicine, date of prescription, original Japanese code of medical procedure, date of any procedures, original Japanese device code, and date of device used). Furthermore, the DPC database contains information on ambulance use, hospitalisation within 6 months, purpose of admission, body mass index (BMI) at admission, and activities of daily living (ADL) measured by the Barthel Index at admission [2] as well as the disease-specific severity information, including the Japan Coma Scale (JCS) [3], systolic blood pressure (SBP) of patients with heart failure, and New York Heart Association (NYHA) classification of heart failure [4]. Moreover, the DPC database includes information on patient outcomes, such as in-hospital death, ADL at discharge, and rehospitalisation within 90 days. However, the database does not contain medical imaging data or specific test results (e.g., left ventricular ejection fraction).

We extracted information from the DPC database of patients who were discharged between April 2018 and March 2020.

#### Supplement 2: Distributed representation learning process

We first converted the information included in the DPC database into a complex of tokens created by concatenating the variable name (key) and value. Some variables, such as the ICD10 codes, are assigned specific classification rules. The first letter of an ICD10 code indicates the chapter of its code, the second letter indicates a large category, and the third and fourth (or fifth) codes indicate detailed categories. As diseases mapped in the same large category have a similar character to the disease, we added larger category tokens of the ICD10 code to the complex of tokens. The details of this process are presented in Supplemental Figure 1.

According to previous studies [5,6], we removed duplicates of tokens of each inpatient and created a ‘sentence’ by concatenating all tokens of each inpatient in random order, separated by a space. A distributed representation of each token was obtained using the word2vec module in the GenSim library in Python [7]. We set the learning mode as CBOW and the window size to 10. Tokens that occurred 25 times or fewer were neglected, and the number of dimensions of the distributed representation was set to 200.

References

1. Yasunaga H. Real world data in Japan: Chapter II. The diagnosis procedure combination database. Ann Clin Epidemiol. 2019;1:76-9.

2. Duffy L, Gajree S, Langhorne P, Stott DJ, Quinn TJ. Reliability (inter-rater agreement) of the Barthel index for assessment of stroke survivors: systematic review and meta-analysis. Stroke. 2013;44:462-8.

3. Shigematsu K, Nakano H, Watanabe Y. The eye response test alone is sufficient to predict stroke outcome-reintroduction of Japan Coma Scale: A cohort study. BMJ Open. 2013;3:e002736.

4. White PD, Myers MM. The classification of cardiac diagnosis. JAMA. 1921;77:1414-5.

5. Beam AL, Kompa B, Schmaltz A, Fried I, Weber G, Palmer N, et al. Clinical concept embeddings learned from massive sources of multimodal medical data. Pac Symp Biocomput. 2020;25:295-306.

6. Feng Y, Min X, Chen N, Chen H, Xie X, Wang H, et al. Patient outcome prediction via convolutional neural networks based on multi-granularity medical concept embedding. IEEE Int Conf Bioinform Biomed BIBM. 2017. [cited Nov 8 2023] Available from: http://ieeexplore.ieee.org/document/8217753/.

7. Rehurek R, Sojka P. Gensim–python framework for vector space modelling; 2. NLP Cent Fac Inform Masaryk Univ Brno Czech Repub. 2011:3.


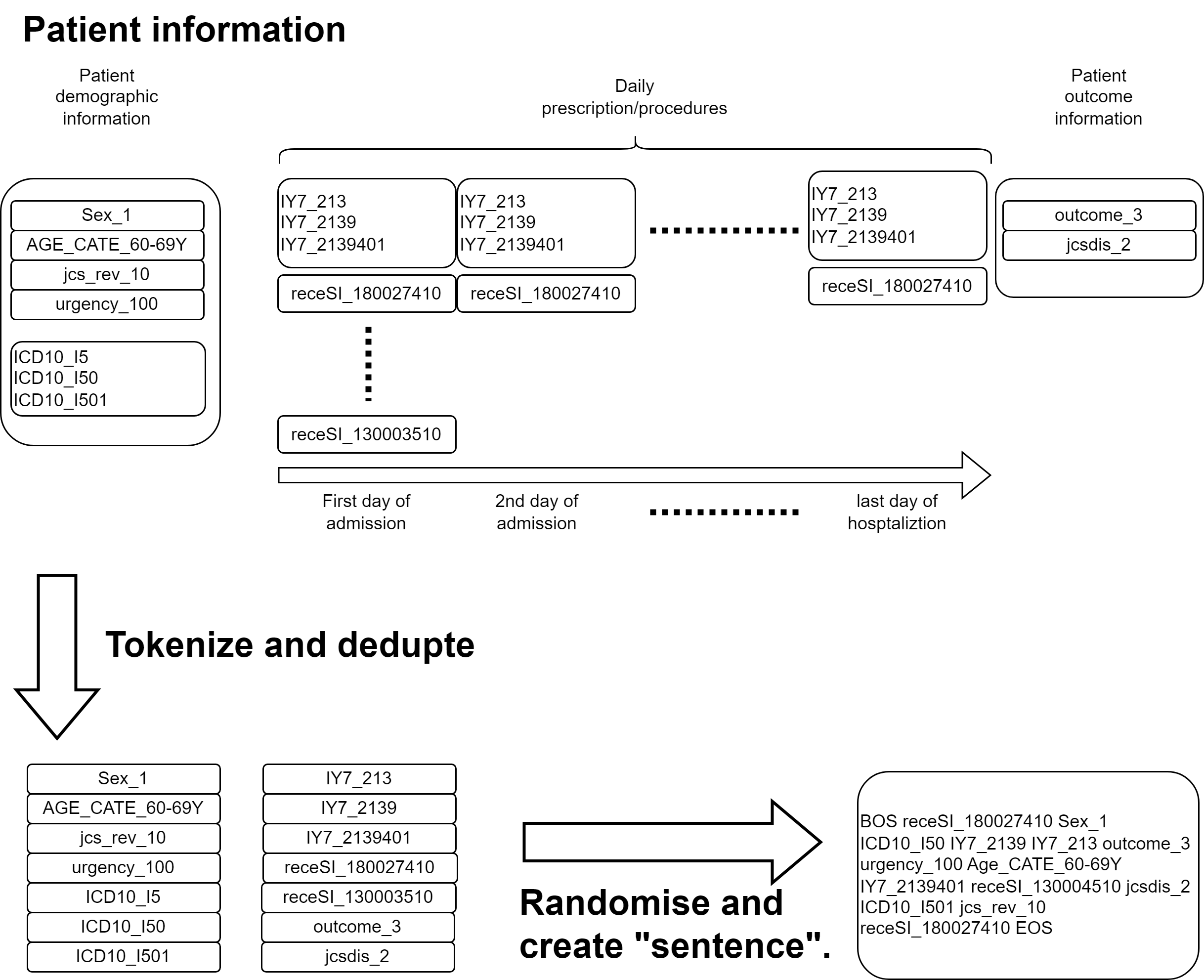


Supplementary Figure S2-1: This figure shows the process of converting the information included in the DPC database into a sentence.
1. All the patient information was converted into a complex of tokens. Each token was constructed as the concatenated information of type and value with an underbar letter.

2. We created a sub-token for information that had a coding rule. For example, we added a chapter token and a large code category token for the ICD10 code. For left ventricular failure (I501), we added, “I” and “I5” tokens for “I501.”

3. We concatenated a complex of tokens for each inpatient with a space letter and created a ‘sentence’.

#### Supplementary Table S1: Patient demographic information of the real-world cohort

| **Characteristics** | **0**, *n* = 229,298 | **1**, *n* = 37,289 | SMD |
| --- | --- | --- | --- |
| **Age (years)** | 81 (12) | 82 (11) | −0.080 |
| **Sex** |  |  | 0.028 |
| Male | 118,019 (51%) | 18,670 (50%) |  |
| Female | 111,279 (49%) | 18,619 (50%) |  |
| **BMI (**kg/m^2^**)** |  |  | 0.074 |
| 18.5–24.9 | 122,547 (53%) | 20,413 (55%) |  |
| 25.0–29.9 | 39,733 (17%) | 6,694 (18%) |  |
| ≥30.0 | 21,595 (9.4%) | 3,413 (9.2%) |  |
| <18.5 | 39,220 (17%) | 6,134 (16%) |  |
| Missing category | 6,203 (2.7%) | 635 (1.7%) |  |
| **NYHA** |  |  | 0.073 |
| 0 (Unclassifiable) | 2,225 (1.0%) | 230 (0.6%) |  |
| 1 (Class 1) | 2,662 (1.2%) | 371 (1.0%) |  |
| 2 (Class 2) | 9,988 (4.4%) | 1,958 (5.3%) |  |
| 3 (Class 3) | 16,861 (7.4%) | 3,149 (8.4%) |  |
| 4 (Class 4) | 12,135 (5.3%) | 1,892 (5.1%) |  |
| Missing category | 185,427 (81%) | 29,689 (80%) |  |
| **ADL at admission** |  |  | 0.042 |
| <60 | 94,465 (41%) | 15,013 (40%) |  |
| ≥60 | 101,122 (44%) | 16,224 (44%) |  |
| Missing category | 33,711 (15%) | 6,052 (16%) |  |
| **Japan Coma Scale** |  |  | 0.079 |
| 0 | 191,836 (84%) | 31,546 (85%) |  |
| 1 | 17,082 (7.4%) | 2,861 (7.7%) |  |
| 10 | 2,854 (1.2%) | 350 (0.9%) |  |
| 100 | 484 (0.2%) | 40 (0.1%) |  |
| 2 | 8,058 (3.5%) | 1,396 (3.7%) |  |
| 20 | 762 (0.3%) | 71 (0.2%) |  |
| 200 | 468 (0.2%) | 29 (<0.1%) |  |
| 3 | 6,703 (2.9%) | 910 (2.4%) |  |
| 30 | 727 (0.3%) | 54 (0.1%) |  |
| 300 | 324 (0.1%) | 32 (<0.1%) |  |
| **Weekend admission** | 15,732 (6.9%) | 2,647 (7.1%) | −0.009 |
| **Hospitalization within 6 months** | 138,010 (60%) | 21,668 (58%) | 0.042 |
| **SBP** |  |  | 0.131 |
| 1 (<100 mmHg) | 21,393 (9.3%) | 3,124 (8.4%) |  |
| 2 (100-140 mmHg) | 99,345 (43%) | 17,187 (46%) |  |
| 3 (>140 mmHg) | 62,431 (27%) | 11,182 (30%) |  |
| Missing category | 46,129 (20%) | 5,796 (16%) |  |
| **Smoking status** |  |  | 0.010 |
| 0 (Non-smoker) | 141,667 (62%) | 23,161 (62%) |  |
| 1 (Smoker) | 87,625 (38%) | 14,128 (38%) |  |
| Missing category | 6 (<0.1%) | 0 (0%) |  |
| **Hypertension** | 119,463 (52%) | 22,289 (60%) | −0.155 |
| **Diabetes mellitus** | 61,802 (27%) | 10,259 (28%) | −0.013 |
| **Chronic renal failure** | 45,803 (20%) | 6,902 (19%) | 0.037 |
| **Chronic liver disease** | 3,847 (1.7%) | 530 (1.4%) | 0.021 |
| **Chronic respiratory disease** | 9,245 (4.0%) | 1,437 (3.9%) | 0.009 |
| **Anaemia** | 21,483 (9.4%) | 3,436 (9.2%) | 0.005 |
| **Stroke** | 5,222 (2.3%) | 767 (2.1%) | 0.015 |
| **Cancer** | 17,887 (7.8%) | 2,391 (6.4%) | 0.054 |
| **Myocardial infarction** | 4,056 (1.8%) | 624 (1.7%) | 0.007 |
| **Dilated cardiomyopathy** | 6,526 (2.8%) | 1,003 (2.7%) | 0.010 |
| **Beta-blocker** | 32,556 (14%) | 5,950 (16%) | −0.049 |
| **Renin**–**angiotensin system inhibitor** | 42,087 (18%) | 7,626 (20%) | −0.053 |
| **Mineralocorticoid receptor antagonist** | 37,486 (16%) | 7,383 (20%) | −0.090 |
| **Tolvaptan** | 34,736 (15%) | 7,062 (19%) | −0.101 |
| **Intravenous inotropic agent** | 20,566 (9.0%) | 3,971 (11%) | −0.057 |
| **Intravenous nitrate** | 18,732 (8.2%) | 3,823 (10%) | −0.072 |
| **Intravenous furosemide** | 107,888 (47%) | 20,395 (55%) | −0.153 |
| **Intravenous carperitide** | 36,955 (16%) | 8,092 (22%) | −0.143 |
| **Respiratory support** | 119,630 (52%) | 20,279 (54%) | −0.044 |
| **Haemodialysis** | 3,978 (1.7%) | 272 (0.7%) | 0.091 |
| **ICU stay within 2 days after admission** | 4,966 (2.2%) | 1,295 (3.5%) | −0.079 |

ADL: activities of daily living; BMI: body mass index; ICU: intensive care unit; NYHA: New York Heart Association Classification of heart failure; SBP: systolic blood pressure; SMD: standardised mean difference

#### Supplement 3: Study variables

Based on a previous study, we collected patient information from the DPC database. We used patient background information (age, sex, previous hospitalisation within 6 months of the index hospitalisation, and weekend hospitalisation) and diagnoses at admission (with or without the following diagnoses: hypertension, diabetes mellitus, chronic renal failure, chronic liver disease, chronic respiratory disease, anaemia, stroke, cancer, myocardial infarction, and dilated cardiomyopathy). Additionally, beta-blockers, renin–angiotensin system inhibitors, mineralocorticoid receptor antagonists, tolvaptan, intravenous inotropic agents, intravenous nitrates, intravenous furosemide, and intravenous carperitide were prescribed on the first day after admission. The following medical procedures were performed on the first day of admission: mechanical ventilation, dialysis, and admission to the intensive care unit. From the discharge summary records, we obtained detailed information of patients with heart failure (HF) including ADL at admission measured using the Barthel index score, systolic blood pressure (categorised into <100, 100–140, and >140mmHg), BMI, smoking status, NYHA, and JCS. Furthermore, we used diagnosis and medical practice information obtained on the first day of admission.

#### Supplement 4: Details of the prognostic model

*Patient population*

For model derivation and validation, we used the entire heart failure population included in this study.

*Model derivation method*

We constructed a logistic regression model as a prognostic model using variables, including disease severity, with reference to previous studies. We set the composite outcome of in-hospital death and dependency on ADL at discharge as the predicted target outcome. Explanatory variables used in the prognostic model included background information (i.e., age, sex, previous hospitalisation within 6 months of the index hospitalisation, and weekend hospitalisation) and admission diagnoses (with or without the following diagnoses: hypertension, diabetes mellitus, chronic renal failure, chronic liver disease, chronic respiratory disease, anaemia, stroke, cancer, myocardial infarction, and dilated cardiomyopathy). The following medical procedures and drug prescriptions on the first day of admission were included in the prediction model: beta-blockers, renin–angiotensin system inhibitors, mineralocorticoid receptor antagonists, tolvaptan, intravenous inotropes, intravenous nitrates, intravenous furosemide, intravenous carperitide, ventilator use, dialysis, and admission to the intensive care unit. Moreover, we included the following detailed information about patients with heart failure as explanatory variables: ADL at admission measured by the Barthel Index, systolic blood pressure (categorised into <100, 100–140, and >140mmHg), BMI, smoking status, NYHA, and JCS.

*Model validation method*

Model discrimination was assessed using the area under the receiver operating characteristic (ROC) curve. We generated a calibration curve and confirmed the correlation between the actual and predicted outcome probabilities.

*Model detail*

**Supplementary Table S4-1.** Details of the prognostic model

| **Characteristic** | **Coefficients** | **95% CI** | ***P*-value** |
| --- | --- | --- | --- |
| **Age (years)** | 0.06 | 0.06–0.06 | <0.001 |
| **Sex** |  |  |  |
| Male | — | — |  |
| Female | 0.08 | 0.06–0.11 | <0.001 |
| **BMI** |  |  |  |
| <18.5 | — | — |  |
| ≥30.0 | −0.26 | −0.31 to −0.21 | <0.001 |
| 18.5–24.9 | −0.43 | −0.46 to −0.40 | <0.001 |
| 25.0–29.9 | −0.55 | −0.59 to −0.51 | <0.001 |
| Missing category | 0.28 | 0.21–0.35 | <0.001 |
| **Hypertension** | −0.38 | −0.40 to −0.35 | <0.001 |
| **Diabetes mellitus** | 0.00 | −0.02 to 0.03 | 0.8 |
| **Chronic renal failure** | 0.16 | 0.13–0.19 | <0.001 |
| **Chronic liver disease** | 0.13 | 0.03–0.22 | 0.007 |
| **Chronic respiratory disease** | −0.07 | −0.13 to −0.01 | 0.027 |
| **Anemia** | −0.02 | −0.06 to 0.02 | 0.3 |
| **Stroke** | 0.40 | 0.33–0.48 | <0.001 |
| **Cancer** | 0.20 | 0.16–0.25 | <0.001 |
| **Myocardial infarction** | 0.00 | −0.09 to 0.08 | >0.9 |
| **Dilated cardiomyopathy** | −0.11 | −0.20 to −0.02 | 0.023 |
| **Smoking status** |  |  |  |
| 0 (Non-smoker) | — | — |  |
| 1 (Smoker) | −0.15 | −0.17 to −0.12 | <0.001 |
| Missing category | −1.2 | −4.2 to 0.80 | 0.3 |
| **Hospitalization within 6 months** | 0.07 | 0.04–0.09 | <0.001 |
| **NYHA** |  |  |  |
| 0 (Unclassifiable) | — | — |  |
| 1 (Class 1) | −0.60 | −0.77 to −0.44 | <0.001 |
| 2 (Class 2) | −0.74 | −0.87 to −0.61 | <0.001 |
| 3 (Class 3) | −0.59 | −0.72 to −0.47 | <0.001 |
| 4 (Class 4) | −0.35 | −0.48 to −0.23 | <0.001 |
| Missing category | −0.42 | −0.54 to −0.30 | <0.001 |
| **SBP** |  |  |  |
| 1 (<100 mmHg) | — | — |  |
| 2 (100-140 mmHg) | −0.44 | −0.48 to −0.40 | <0.001 |
| 3 (>140 mmHg) | −0.62 | −0.66 to −0.57 | <0.001 |
| Missing category | −0.40 | −0.45 to −0.35 | <0.001 |
| **ADL at admission** |  |  |  |
| <60 | — | — |  |
| ≥60 | −2.5 | −2.5 to −2.5 | <0.001 |
| Missing category | −1.4 | −1.5 to −1.4 | <0.001 |
| **Japan Coma Scale** |  |  |  |
| 0 | — | — |  |
| 1 | 0.50 | 0.46–0.53 | <0.001 |
| 10 | 1.3 | 1.2–1.4 | <0.001 |
| 100 | 1.4 | 1.2–1.7 | <0.001 |
| 2 | 0.93 | 0.87–0.98 | <0.001 |
| 20 | 1.4 | 1.2–1.6 | <0.001 |
| 200 | 1.8 | 1.5–2.1 | <0.001 |
| 3 | 1.4 | 1.4–1.5 | <0.001 |
| 30 | 1.6 | 1.4–1.9 | <0.001 |
| 300 | 1.1 | 0.8–1.4 | <0.001 |
| **Weekend admission** | −0.02 | −0.06 to 0.03 | 0.5 |
| **Beta-blocker** | −0.20 | −0.24 to −0.17 | <0.001 |
| **Renin–angiotensin system inhibitor** | −0.28 | −0.32 to −0.25 | <0.001 |
| **Mineralocorticoid receptor antagonist** | −0.01 | −0.05 to 0.02 | 0.4 |
| **Tolvaptan** | 0.03 | −0.01 to 0.06 | 0.14 |
| **Intravenous inotropic agent** | 0.22 | 0.18–0.27 | <0.001 |
| **Intravenous nitrate** | −0.33 | −0.37 to −0.28 | <0.001 |
| **Intravenous furosemide** | −0.15 | −0.18 to −0.13 | <0.001 |
| **Intravenous carperitide** | −0.10 | −0.13 to −0.07 | <0.001 |
| **Respiratory support** | 0.12 | 0.09–0.14 | <0.001 |
| **Haemodialysis** | −0.19 | −0.29 to −0.09 | <0.001 |
| **ICU stay within 2 days after admission** | −0.14 | −0.21 to −0.06 | <0.001 |

BMI, body mass index; CI, confidence interval; NYHA, New York Heart Association Classification of heart failure; SBP, systolic blood pressure; ADL, activities of daily living.

*Model validation result*

Area under the ROC curve was: 0.8678 (0.8620–0.8684) and the model showed good calibration (Supplementary Figure S4-1).


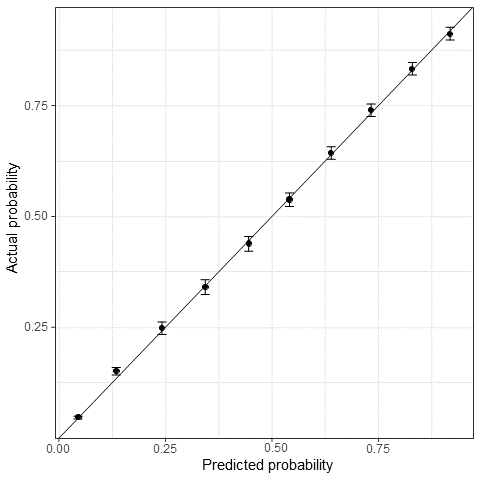


Supplementary Figure S4-1: Calibration curve


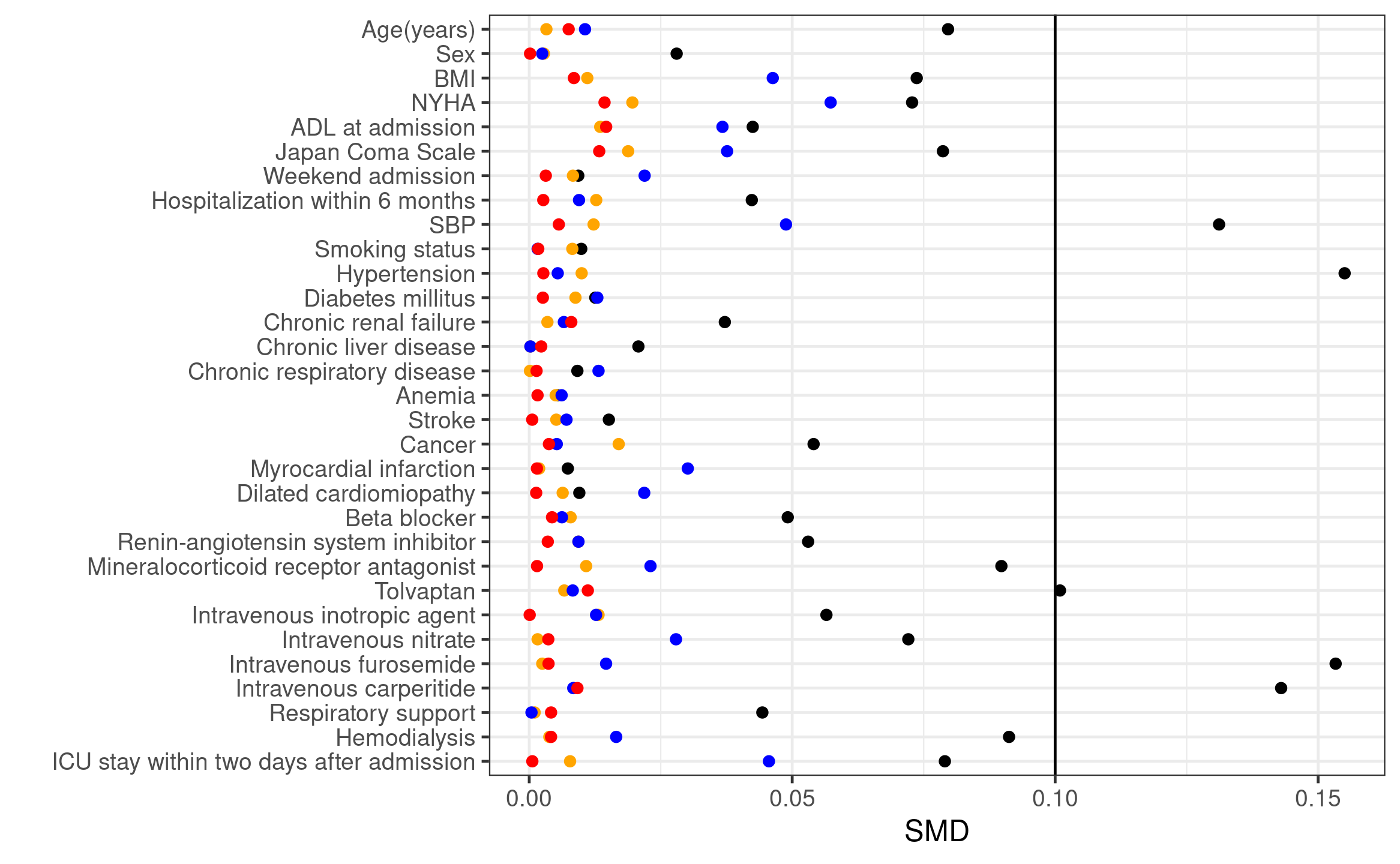


#### Supplementary Figure S1. Covariate balances for each risk-adjustment model

ADL: activities of daily living; BMI: body mass index; ICU: intensive care unit; NYHA: New York Heart Association Classification of heart failure; SBP: systolic blood pressure; SMD: standardised mean difference


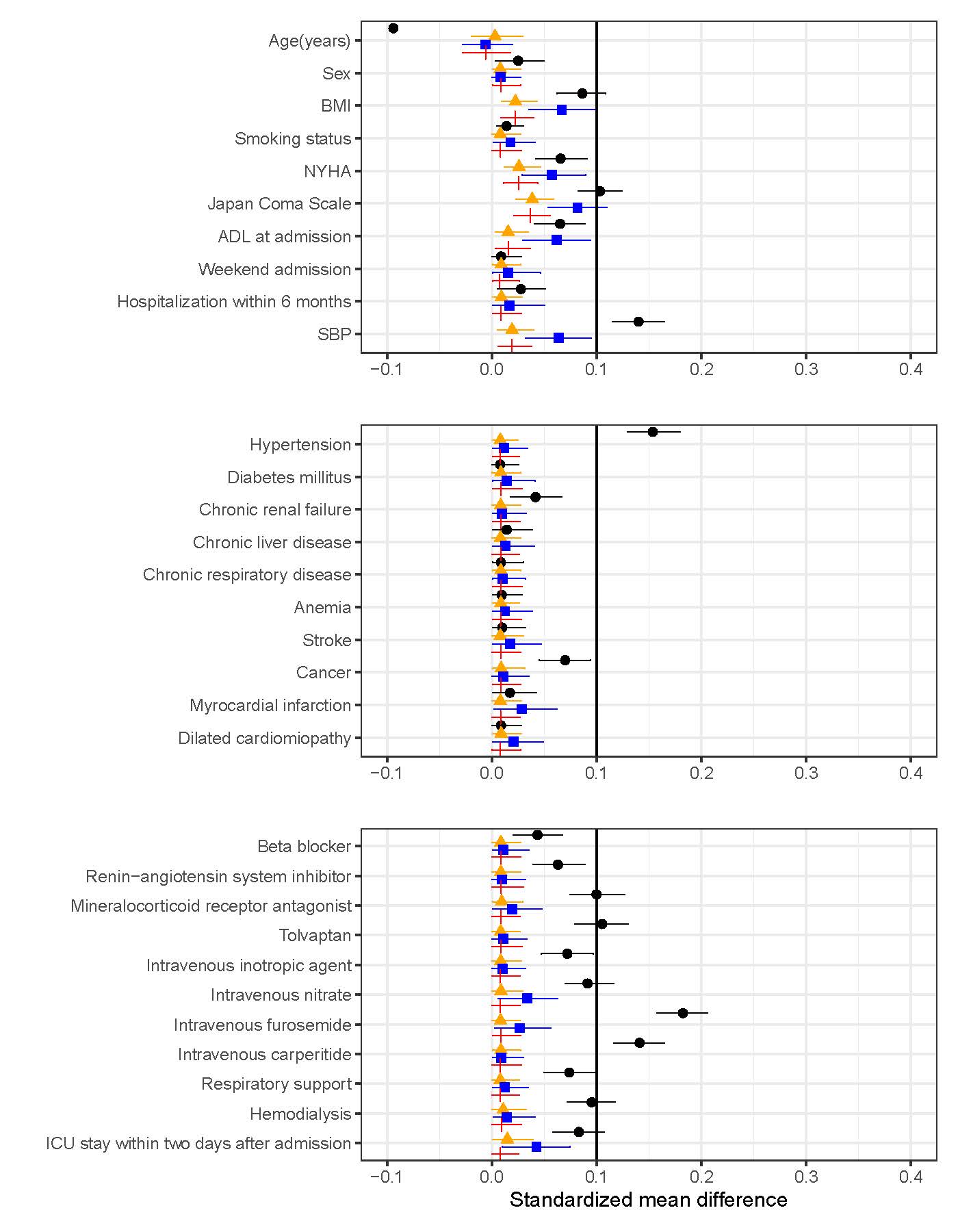


Supplementary Figure S2: The covariate balance between the control and exposure groups measured with standardised mean difference and its 95% confidence intervals (CI). Dots indicate point estimates and bars indicate 95% CI. The dots and bars are black, orange, blue, and red for models 1, 2, 3, and 4, respectively.

#### Supplement 5: Covariate balance of variables.
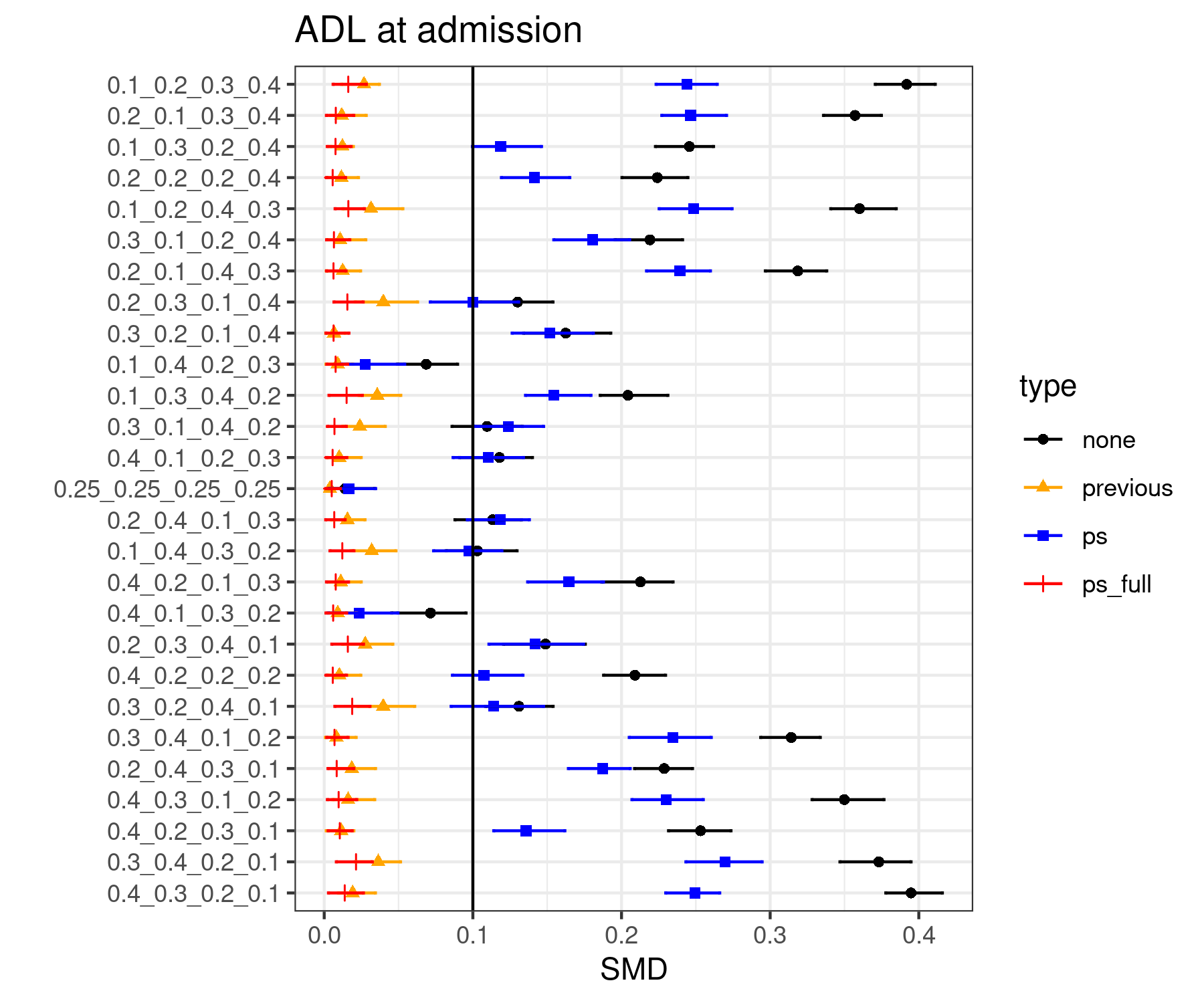


Supplementary Figure S5-1: Covariate balance for ADL at admission


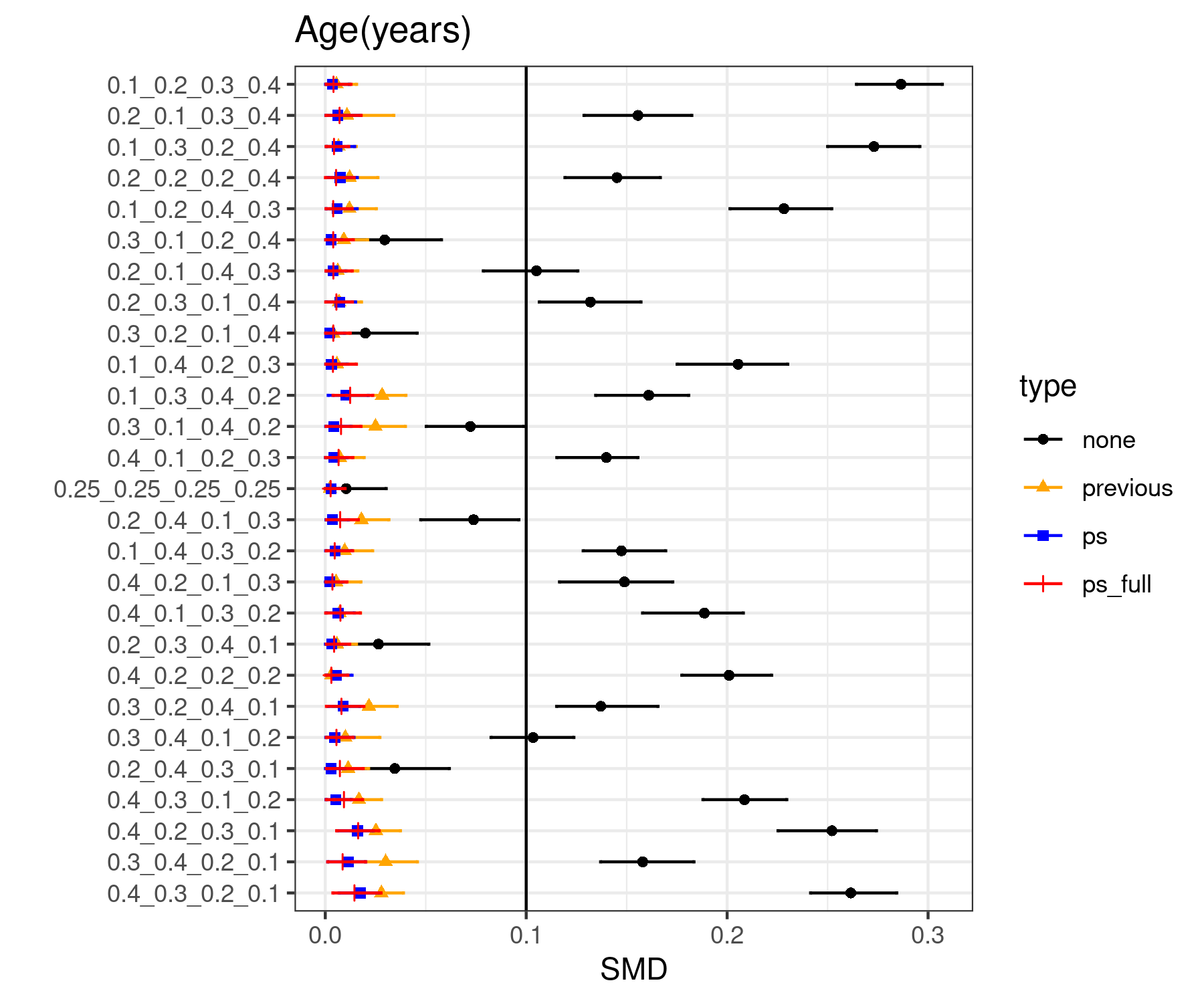


Supplementary Figure S5-2: Covariate balance for age.


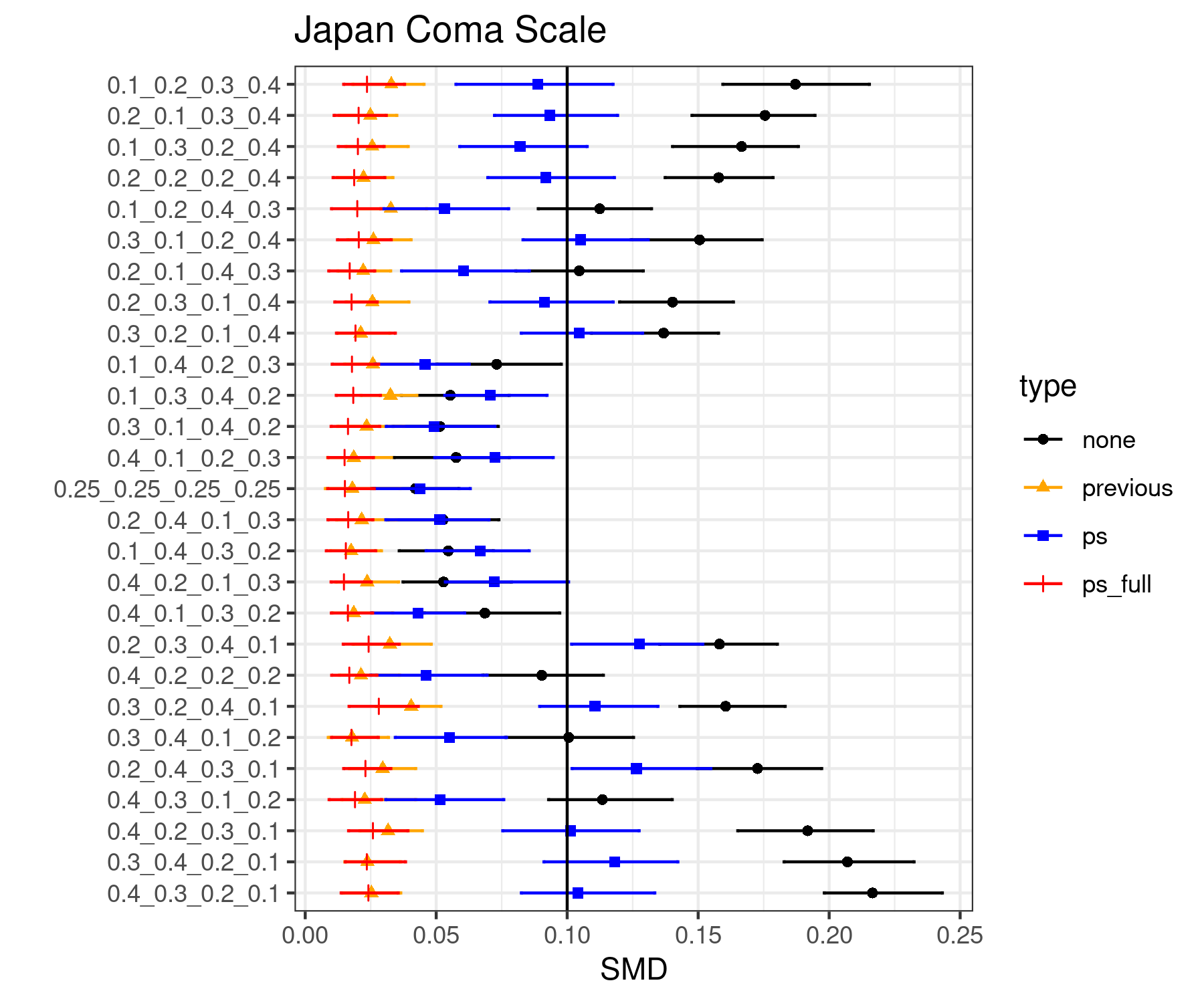


Supplementary Figure S5-3: Covariate balance for the Japan Coma Scale.

#### Supplement 6

**Supplementary Table S6-1. Drugs close to (associated with) heart failure (I50) in vector representation**

| Label category | Key | Similarity | Name |
| --- | --- | --- | --- |
| IY7 | 2139005 | 0.5916612 | Furosemide |
| IY7 | 2139008 | 0.5888991 | Azosemide |
| IY7 | 2149032 | 0.5863839 | Carvedilol |
| IY7 | 2179400 | 0.5828117 | Carperitide (Genetic Recombination) |
| IY7 | 2144002 | 0.5709956 | Enalapril maleate |

**Supplementary Table S6-2. Medical procedures close to (associated with) heart failure (I50) in vector representation**

| Label category | Key | Similarity | Name |
| --- | --- | --- | --- |
| receSI | 160162350 | 0.5687901 | Blood test (BNP) |
| receSI | 160072510 | 0.547079 | Echocardiography (transthoracic echocardiography) |
| receSI | 180027410 | 0.5279201 | Cardiovascular rehabilitation fee (1) |
| receSI | 160064610 | 0.4987158 | Cardiac catheterisation (right heart) |
| receSI | 160062010 | 0.4981422 | Biochemical tests (2) Decision fee |
